# Open Coronary Endarterectomy of Left Anterior Descending Artery—Case Report and Review of Literature

**DOI:** 10.3390/jcdd9030083

**Published:** 2022-03-13

**Authors:** Mircea Robu, Diana Romina Marian, Ecaterina Lazăr, Răzvan Radu, Cristian Boroș, Andra Sibișan, Cristian Voica, Marian Broască, Daniela Gheorghiță, Horațiu Moldovan, Vlad Anton Iliescu

**Affiliations:** 1 Department of Cardiovascular Surgery, Prof. Dr. C.C.Iliescu Emergency Institute for Cardiovascular Diseases, 022328 Bucharest, Romania; mircearb@yahoo.com (M.R.); diana.carabas@yahoo.com (D.R.M.); ecaterinaioanalazar@gmail.com (E.L.); vladanton.iliesc@gmail.com (V.A.I.); 2 Cardiology Department, Prof. Dr. C.C.Iliescu Emergency Institute for Cardiovascular Diseases, 022328 Bucharest, Romania; razvanir@yahoo.com; 3 Anesthesia and Intensive Care Department, Prof. Dr. C.C.Iliescu Emergency Institute for Cardiovascular Diseases, 022328 Bucharest, Romania; boros_c@yahoo.com; 4 Department of Cardiovascular Surgery, Clinical Emergency Hospital Bucharest, 014461 Bucharest, Romania; andrasibisan@yahoo.com (A.S.); cristianvoica@yahoo.com (C.V.); marian.broasca@gmail.com (M.B.); 5 Faculty of Materials Science and Engineering, Politehnica University of Bucharest, 060042 Bucharest, Romania; daniela.mgm8@gmail.com; 6 Faculty of Medicine, Carol Davila University of Medicine and Pharmacy, 050474 Bucharest, Romania

**Keywords:** coronary endarterectomy, coronary bypass, arterial grafts, ischemic heart disease

## Abstract

Coronary endarterectomy (CE) emerged as a necessity to achieve complete surgical myocardial revascularization in patients with diffusely diseased coronary arteries and it also serves as aid to coronary bypass grafting (CABG). The safety and postoperative prognosis of this procedure are still matters of debate. There are no clear preoperative indications, a standard technique has not yet been established as gold standard and the postoperative management differs depending on each institution. CE of the left anterior descending artery (LAD) is technically challenging and potentially hazardous with high risk of postoperative myocardial infarction. In this article, we describe the open technique for CE of the LAD with its specific details, which we believe could be the safest and the best reproductible option. To better understand the profile of a patient requiring such a procedure we present the case of a 73-years old male with diffused coronary artery disease (CAD) and a short review of literature.

## 1. Introduction

Coronary endarterectomy (CE) was initially described in 1957 by Bailey as a technique to achieve complete myocardial revascularization in patients with diffusely diseased coronary arteries [1]. Studies regarding the safety and necessity of this procedure are controversial and a standard technique of CE on the LAD has not yet been established [2,3,4,5,6,7,8,9,10,11,12,13,14,15,16,17,18,19,20,21,22,23,24,25,26,27,28]. There is an increased risk of postoperative myocardial infarction when CE is performed on the LAD with potential fatal consequences [7]. We describe the open technique, the preferred technique used in our center, which has been proven to be safe and reproductible in order to achieve complete myocardial revascularization. This technique was used in our institution as a necessity in patients requiring myocardial revascularization and also combined procedures such as aortic valve replacement with very good results.

## 2. Case Report

**Figure 1:**
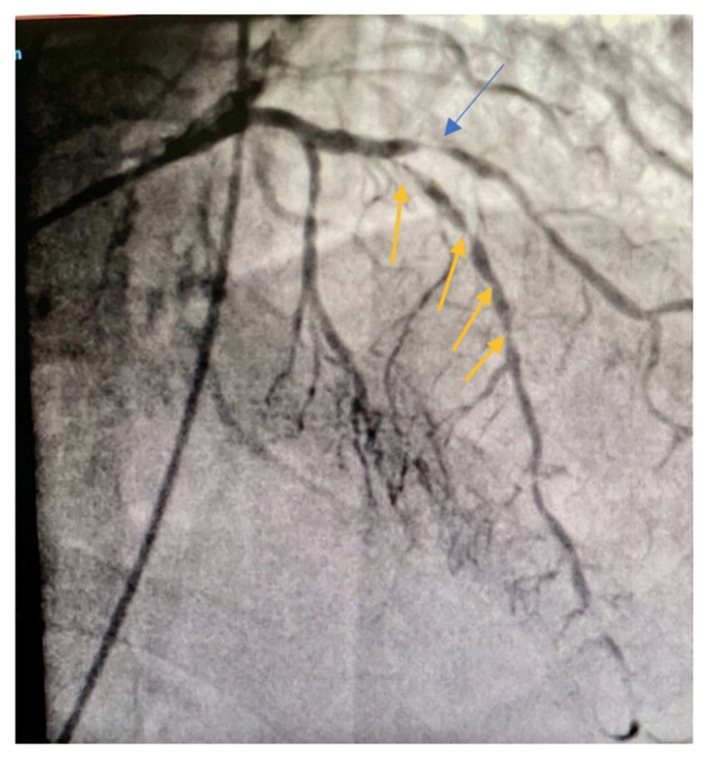
Diffusely diseased LAD artery with multiple severe distal stenosis (orange arrows) and proximal severe stenosis on the diagonal branch (blue arrow).

We present a case of a 76-year-old non-smoker, hypertensive, dislipidemic male, addressed to our service for chest pain. Coronary angiography revealed severe coronary atherosclerosis with 90% stenosis in the second segment of LAD and distal occlusion and a 90% proximal stenosis at the level of the first diagonal (Figure 1).

Transthoracic echocardiography revealed degenerative aortic valve disease with severe stenosis (mean gradient of 45 mmHg) and mild regurgitation with good biventricular function. Preoperative Doppler ultrasound of the carotid arteries showed a 60% stenosis on the left internal carotid artery. Blood panel revealed no significant findings other than dyslipidemia.

Patient was scheduled for aortic valve replacement (AVR) with a biological prosthesis and double coronary artery bypass using the internal thoracic artery (ITA) (skeletonized) and a venous graft with extracorporeal circulation. Median sternotomy and standard cannulation for AVR were employed and the heart was arrested using cold blood cardioplegia in the anterograde and retrograde manner. Two separate doses of cardioplegia were administered later in the retrograde fashion. First, the distal anastomosis between a venous graft and the first diagonal was realized using a continuous Prolene 8.0 suture. After completion, the ascending aorta was transversely incised and AVR was performed using a 25 mm Edwards Lifesciences pericardial valve bioprosthesis. Next, we evaluated the LAD for a suitable place for a distal anastomosis but the vessel was severely calcified through all its length including the distal portion. Serial anastomosis on LAD could not be performed. We decided that CE was the only way for this particular LAD in order to achieve complete myocardial revascularization.

### Our Technique

**Figure 2:**
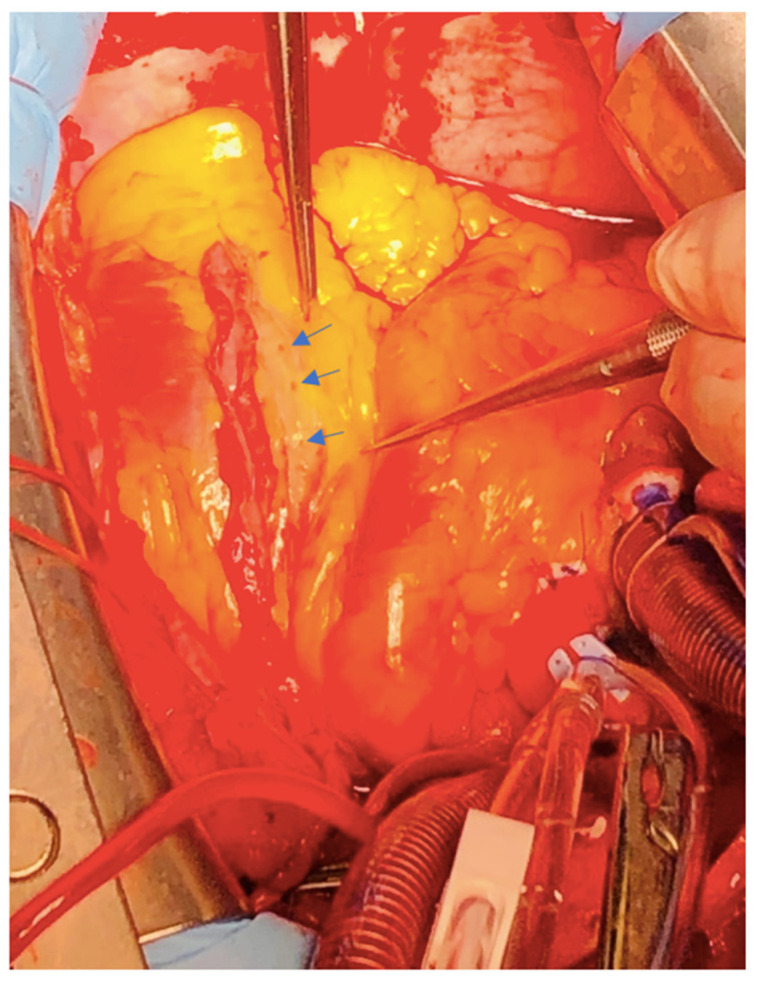
Adventitia of the left anterior descending artery after plaque extraction. Several origins of septal branches can be observed (blue arrows). Left ITA harvested in a skeletonized fashion is prepared for termino-lateral anastomosis.

The technique that we opted for is the open one [1]. Longitudinal arteriotomy (Figure 2) was started just 1 cm below the first diagonal and advanced on the entire length of the LAD in a region where the walls of the vessel appeared normal.

A 1.5 mm probe was inserted distally in the native remaining coronary artery to assess the permeability. If the probe doesn’t progress all the way to the apex, the arteriotomy needs to be extended even with a normal aspect of the coronary walls in order to eliminate all the stenotic areas from the vessel. Using this technique, the arteriotomy was extended at about 2 cm proximal to the apex of the heart. CE was performed using a fine spatula and fine scissors used for internal thoracic artery harvesting starting distally and advancing to the apex. Only the LAD adventitia was left. 

**Figure 3:**
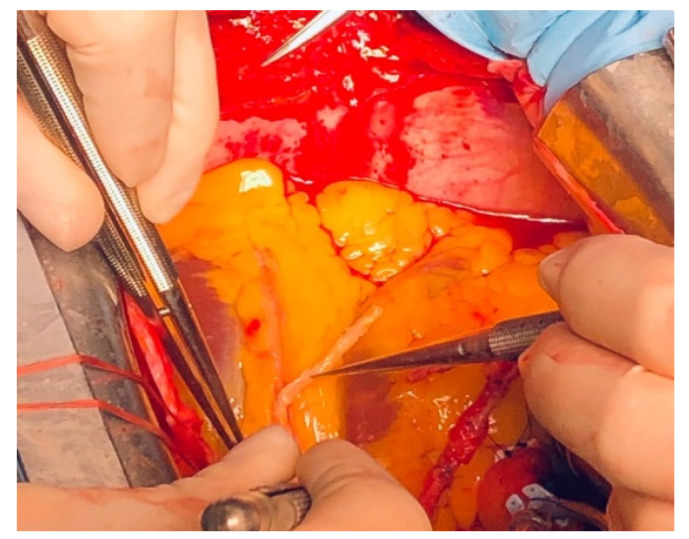
Extracting the plaque “en bloc”.

We identified several septal branches (Figure 2) which were dissected 2–3 mm in depth and sectioned with fine scissors. Extraction of the LAD plaque was done “en bloc”, maintaining the same plane of dissection with limited manipulation of the adventitia (Figure 3). 

We then thoroughly washed with saline solution in order to clear all the possible debris. The left ITA was placed parallel to the arteriotomy and longitudinal arteriotomy was performed preparing for the termino-lateral anastomosis. The anastomosis was done in a standard parachute technique using Prolene 8.0 continuous suture. 

**Figure 4:**
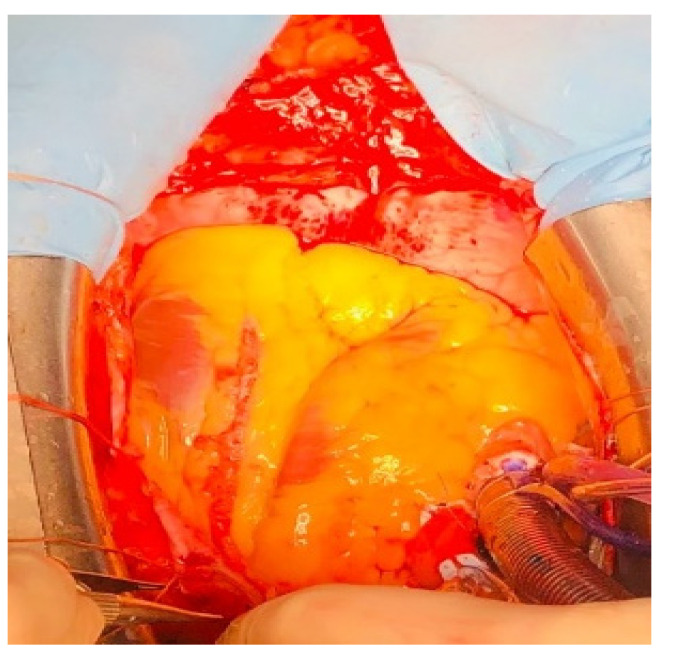
Final result after declamping de left internal mammary artery.

Before completing the anastomosis, another saline wash was performed before declamping the ITA, then the graft was declamped and blood was allowed to flow outside the anastomosis before completing the anastomosis (Figure 4).

Cardiopulmonary bypass time was 167 min, aortic cross clamp time was 137 min. Weening of cardiopulmonary bypass (CPB) was uneventful, patient required small doses of vasopressor support without inotrope or intra-aortic balloon pump (IABP) insertion. Intraoperative flow measurements were done to evaluate graft patency. Flow at the level of ITA graft was 64.7 mL/min with a pulsability index of 4.09 and diastolic pattern specific for the left ventricle (Figure 4). We also recommend thorough hemostasis of the mediastinal wound and sternum to reduce the risk of bleeding and large opening and drainage of both pleural cavities to reduce the risk of cardiac tamponade in the immediate postoperative faze because antiplatelet and anticoagulation therapy is introduced within 4–6 h after surgery.

Patient had an uneventful recovery in the intensive care unit (ICU), was transferred on the surgical ward on the second day and left the hospital in the 7th day without any complications according to our institution protocol. Patient was extubated 10 h after surgery. Double antiplatelet treatment with Aspirin and Clopidogrel was initiated 4 h after ICU admission and heparin infusion was started 6 h postoperatively. Postoperative electrocardiogram (EKG) was the same as the preoperative one, showing normal sinus rhythm with narrow QRS complexes without any ST-T modifications. A brief episode of atrial fibrillation was converted to sinus rhythm using Amiodarone. No other arrhythmias were observed. Cardiac enzymes (CK/CK-MB) shifted from 445/72 to 639/38 to 131/17. Minimal vasopressor support was required on the first day. Serial transthoracic echocardiographic studies showed good biventricular function without any areas of dyskinesia with a normal functioning aortic prosthesis and without any pericardial effusions. Chest drainage was the following: 700 mL in the first 24 h, 150 mL from mediastinum and 325 mL from both pleural the next 24 h. Chest drains were removed on the third day. Heparin infusion was combined with oral antivitamin K (AVK) beginning on the 2nd day with a target INR between 2.5–3.

Dual antiplatelet and oral AVK therapy continued for 3 moths after which only antiplatelet therapy with Clopidogrel was recommended. 1-, 3- and 6-months cardiologist check-ups found the patient in good clinical condition without chest pain and good effort tolerance, blood tests revealed a mild anemic syndrome. EKG tracings showed normal sinus rhythm without any ST-T elevation, transthoracic echocardiography showed normal biventricular function with a normal functioning aortic valve bioprosthesis. 

## 3. Discussions

Coronary endarterectomy was first described in 1957 by Bailey [2], as a method for revascularization for general use and has now been rendered of use for severely calcified coronary arteries [2]. The importance of this technique is the possibility of total myocardial revascularization as opposed to an otherwise incomplete revascularization associated with a higher early mortality rate, recurrent angina and myocardial infarction [3,4]. This procedure remains controversial to this day because higher incidence of postoperative myocardial infarction has been reported in the early postoperative period due to acute thrombosis after exposing the coronary adventitia to circulation [3,4].

Regarding CE on LAD, the general opinion is that this procedure is technically demanding, given the fragility of the atherosclerotic plaque with high risk of rupture and the origin of the diagonal and septal branches arising from different planes, thus putting them at risk of tearing during plaque extraction [7]. The procedure can be hazardous considering the area of myocardium at risk in case of acute thrombosis in the early postoperative period. Taking everything into account the need for a standard technique is required. The two possible techniques that have now been described are the open and closed manners. The open technique, that consists in a large arteriotomy and plaque removal with a spatula, is the most preferred one, with better overall results, even better when using only arterial grafting.

There is a lack of consensus regarding preoperative indications of this technique. Several authors have reported using this technique in occluded vessels with multiple stenosis and distal involvement [3,4], while others added the presence of viable myocardium with documented reversable ischemia [3,5].

In some cases, this technique for CE is unplanned, it arises as a necessity, when it remains the only possibility to graft a coronary artery previously viewed as suitable on coronary angiography in order to achieve total myocardial revascularization. More complex procedures involving CE and valvular procedures have also been described, without any influence on overall mortality [15].

Correlation between the site of CE and postoperative mortality is also controversial. CE performed on the right coronary artery is reported to have a low risk for complications while CE on the LAD is associated in some studies with a higher early mortality rate [4,6,7,8]. The choice of grafts has also been a matter for debate. Studies describe both the use of venous and arterial grafts with by far superior results for the latter [9,10,14,15]. Furthermore studies have showed that CE with multiple coronary bypass grafting is followed by a higher rate of postoperative mortality [9]. 

Regarding the technique we used, there are several key points and tips to consider. We opted for a longitudinal arteriotomy and prolonging the arteriotomy up to healthy looking tissue, an aggressive approach, in order to prevent flaps of intimal tissue obstructing the toe of the anastomosis. When dissecting the septal branches, we consider important not to tear these branches but cut them with fine scissors in order to avoid rupture and bleeding. It is also important not to dissect too much of the epicardial fat from the adventitia because this layer provides a structural support for the anastomosis and is very important for future haemostasis. 

Extracting the LAD plaque ‘en bloc’ reduces the risk of plaque rupture while maintaining the integrity of the remaining adventitia. It also minimizes the quantity of calcified debris in the operating field that can cause distal embolization.

Considering that the coronary arteriotomy is several centimeters long, it is important that the incision on the ITA is done carefully in the long axis in order to avoid twisting the artery and compromising the anastomosis flow.

The proximal part of the LAD corresponding to the heel of the anastomosis will be the only part of coronary that will have remaining plaque, therefore passing the 8.0 needle can be problematic. Also, at this region it is important to proper descend the ITA graft without tearing the 8.0 wire or the ITA tissue, because of the difficult passing of the wire through calcified plaque. Bleeding can occur in this region so it’s important to proper fix the graft when descending it. We recommend no more than three steps before descending the ITA graft. After descending the graft, attention is given to the side of the arteriotomy distal to the surgeon. Steps should be taken no more than 1–2 mm apart, whilst being careful to always go through the adventitia.

Always integrate in the suture the epicardial fat adjacent to the adventitia for homeostatic purposes, a step that can have two challenges: the epicardial fat can protrude inside the anastomosis creating the possibility of thrombosis or stenosis and if the epicardial fat is separated from the adventitia during plaque extraction, a ridge can be created between this two planes and if bleeding occurs it can be misleading as blood flows through the ridge away from the actual source.

Another key point regarding constructing the anastomosis is the toe. The same principles apply as for a regular anastomosis with one extra step: the intimal flaps that remain on each side must be included in the anastomosis. If this is not done, intimal flaps can compromise the anastomosis by obstructing the flow to the native coronary. After passing the toe, attention must be given to the arteriotomy closest to the surgeon. Steps on this side should be taken with extra caution in the same manner as for the other side because haemostasis in this region can be problematic. There is a risk of right ventricular rupture after extra hemostatic wires are placed in this region after endarterectomy.

Haemostasis at the level of the anastomosis should be obtained as soon as possible after declamping the graft with the heart still arrested. However, we observed that cardiac contraction starts earlier than normal after CE. If extra stiches are needed, we recommend Prolene 7.0 to be placed only with the arrested heart especially on the side of the right ventricle. In the area of the toe, because of the risk of compromising the stability of the intimal flaps, we recommend only superficial Prolene 8.0 stiches to be passed only through epicardial fat and adventitia of ITA. Concerning haemostasis another problem we encountered was bleeding following the initial haemostasis after declamping the aorta with the heart sitting in its normal position in the pericardium. At this stage an extra stitch should be the last resort because of the risk of compromising the entire anastomosis and worsening the bleeding. If local haemostasis with surgical gauzes cannot be achieved the heart should be arrested for safely placing the extra stitch.

Another aspect that should be discussed is the anticoagulation regimen that follows this procedure. Literature suggests different anticoagulation and antiplatelet regimens [3,5,24,25]. Our clinic opted for double antiplatelet therapy using Aspirin and Clopidogrel and anticoagulation started as soon as possible in the immediate postoperative period and continued for three months, a treatment we believe to be of paramount importance both for reducing the risk of perioperative myocardial infarction and long term complications.

## 4. Conclusions

The profile of a patient that needs to undergo CABG has changed throughout the years considering the development of percutaneous procedures for coronary obstruction, thus leaving to surgery only complex cases that require elaborated surgical procedures.

A key point is not to hesitate to perform CE when this becomes a necessity in order to achieve complete revascularization. We consider that trying to do a simple termino-lateral anastomosis or serial anastomosis (unusable) on a severe calcified LAD and keep the CE as a second option is dangerous, given that LAD supplies blood to a large area of myocardium. While weaning the patient off cardiopulmonary bypass after a faulty anastomosis on the LAD there is a high risk of subendocardial ischemia. Rearresting the heart at this moment and performing CE is hazardous with a high risk of cardiogenic shock and poor prognosis.

## Data Availability

Data available on request.
